# Prematurity and body composition at 6, 18, and 30 years of age: Pelotas (Brazil) 2004, 1993, and 1982 birth cohorts

**DOI:** 10.1186/s12889-021-10368-w

**Published:** 2021-02-09

**Authors:** Caroline Cardozo Bortolotto, Iná S. Santos, Juliana dos Santos Vaz, Alicia Matijasevich, Aluísio J. D. Barros, Fernando C. Barros, Leonardo Pozza Santos, Tiago Neuenfeld Munhoz

**Affiliations:** 1 Federal University of Pelotas (UFPel). Postgraduate Program in Epidemiology, Rua Marechal Deodoro, 1160 - 3° Piso. Bairro Centro, Cep: 96020-220, Pelotas, RS Caixa Postal 464 Brazil; 2 Pontifical Catholic University of Rio Grande do Sul, Program of Pediatrics and Child Health, Porto Alegre, Brazil; 3 Federal University of Pelotas, Faculty of Nutrition, Pelotas, 96010610 Brazil; 4 Faculdade de Medicina FMUSP, Departamento de Medicina Preventiva, Universidade de São Paulo, São Paulo, 01246-903 Brazil; 5 Federal University of Pelotas, International Center for Equity in Health, Graduate Program in Epidemiology, Pelotas, 96020220 Brazil; 6 Catholic University of Pelotas, Medicine School, 96010-280 Pelotas, Brazil; 7 Federal University of Pampa (Unipampa), Nutrition College, Itaqui, 97650-000 Brazil; 8 Federal University of Pelotas, Faculty of Psychology, Pelotas, 96030-001 Brazil

**Keywords:** Body fat, Fat mass, Fat-free mass, Body composition, Body mass index, Preterm, Air displacement plethysmography, Cohort studies

## Abstract

**Background:**

We aimed to investigate the association between preterm birth and body composition at 6, 18, and 30 years of age using data from three population-based birth cohort studies.

**Methods:**

Gestational age (GA), defined by the date of the last menstrual period (categorized in ≤33, 34–36, and ≥ 37 weeks), was gathered in the first 24-h after delivery for all live births occurring in the city of Pelotas, Brazil, in 2004, 1993 and 1982. Body composition was assessed by air-displacement plethysmography. Outcomes included fat mass (FM, kg), percent FM (%FM), FM index (FMI, kg/m^2^), fat-free mass (FFM, kg); percent FFM (%FFM), FFM index (FFMI, kg/m^2^), body mass index (BMI, kg/m^2^ at 18 years in the 1993 cohort and 30 years in the 1982 cohort), and BMI Z-score (at 6 years in the 2004 cohort). We further explored the association of birth weight for GA with body composition indicators and BMI. Crude and adjusted linear regressions provided beta coefficients with 95% confidence intervals (95%CI).

**Results:**

A total of 3036, 3027, and 3369 participants, respectively, from the 2004, 1993, and 1982 cohorts were analyzed. At 6 years, preterm boys (born at 34–36 weeks) presented lower adjusted mean of FM (β = − 0.80 kg, − 1.45;-0.16, *p* = 0.046), %FM (β = − 2.39%, − 3.90;-0.88, *p* = 0.008), FMI (β = − 0.70 kg/m^2^, − 1.13;-0.27, *p* = 0.004) as well as lower FFM (β = − 0.4 kg, − 0.77; − 0.12, *p* = 0.010) and FFMI (β = − 0.3 kg/m^2^, − 0.46;-0.10, *p* < 0.001), and BMI Z-score (β = − 0.69,; − 0.99;-0.40, *p* < 0.001); but higher %FFM (β = 2.4%, 0.87;-3.90, *p* = 0.008), when compared to boys born at term (≥37). At 30 years, FM (15.7 kg, 0.25;31.1, *p* = 0.102) was higher among males born at ≤33 weeks. No association was observed for females from the three cohorts and for 18-year-old males. The association of birth weight for GA with body composition and BMI was not significant in any cohort. At 6 years, SGA boys had lower FFMI than boys AGA.

**Conclusions:**

Our results suggest that preterm birth is associated with decreased body fat and fat-free mass in childhood but higher fat mass in adulthood. Nevertheless, results were only significant for males. SGA boys also showed lower FFMI.

**Supplementary Information:**

The online version contains supplementary material available at 10.1186/s12889-021-10368-w.

## Background

Prematurity is globally recognized as a public health problem since its complications are the leading cause of neonatal and infant deaths in middle- and upper-income countries [1], including Brazil [2–4]. Preterm babies (i.e., babies born before 37 weeks of pregnancy) [5] account for an estimated 15 million births worldwide annually, resulting in approximately 1 million deaths among children under 5 years of age [6]. In comparison to those born at term, preterm babies have worse health outcomes [7] and higher mortality risk [8, 9]. Brazil is the 10th country in the world with the highest absolute number of preterm births [5, 10]. The prevalence ranges from 3.4 to 15.0% according to the region of the country [11].

The development of metabolic functions can be initiated in the fetal stage. Imbalance in intrauterine conditions would trigger an adaptive process aiming at survival in more critical conditions, as stated by the theory of Developmental Origins of Health and Disease (DoHaD) [12]. Through metabolic changes, including reduced insulin sensitivity, preterm birth may increase the risk of adult obesity and noncommunicable diseases, such as diabetes mellitus, arterial hypertension, and coronary heart disease. Studies also suggest that such changes are different in males and females [13, 14].

An individual’s body composition reflects the accumulation of nutrients acquired and retained by the body over time. The investigation of body composition, according to the gestational age (GA) at birth, is scarcely reported in the literature. Studies evaluating the relationship between GA and body composition have shown that individuals born preterm presented higher body fat in childhood, adolescence, and adult life [15–19] and lower fat-free mass at four and between 8 and 12 years of age [20–22] when compared to those born at term.

The 2004, 1993, and 1982 Pelotas Cohort Studies have followed participants since birth and have assessed body composition when they were 6, 18, and 30 years of age, respectively. With this information, we can further understand the role of prematurity on body composition in children, adolescence, and adulthood. Therefore, we aimed to evaluate the association between prematurity and body composition at 6, 18, and 30 years of age in males and females of The Pelotas (Brazil) 2004, 1993, and 1982 Birth Cohort Studies, respectively.

## Methods

### Sample population

Pelotas is a city located in southern Brazil with 328,275 inhabitants and a Human Development Index of 0.739 [23]. In 1982, 1993, and 2004, all hospital-born children in Pelotas were followed within three cohort studies using similar methods [24]. For the current study, we used data collected at birth and follow-up visits at the age of 6, 18, and 30 years, respectively, from the 2004, 1993, and 1982 cohorts. At birth visit, trained field workers interviewed mothers at the maternity hospitals within 24 h after delivery. At 6, 18, and 30-year-old follow-ups, participants were invited to attend a research clinic specially designed for the studies. Details on each study methods are available in previous publications [25–27]. All participants with available data on GA and body composition were included in the present analyses. Multiple births and children with congenital malformations that would interfere with feeding and walking were excluded.

### Gestational age

In 1993 and 2004, the algorithm proposed by the National Center for Health Statistics (NCHS) [28] was used to estimate gestational age (GA). The estimated age was based on the last menstrual period whenever it was consistent with birth weight, length, and head circumference, according to the standard curves for these parameters for each week of gestational age [29]. In case the last menstrual period-based gestational age was unknown or inconsistent, we adopted the clinical maturity estimate based on the Dubowitz method [30]. Dubowitz is determined by the inspection of various physical signs and neurological characteristics that vary with fetal age and maturity and consists of 34 items grouped into six dimensions: tone, type of tone, reflexes, movements, abnormal signs, and behavior. In 1982, GA at birth was calculated based on the date of the last menstrual period, and children whose birth weight was incompatible with standards for the estimated age were considered of unknown GA. All participants born before 37 weeks were considered preterm. For analysis, GA was categorized into three groups (≤33, 34–36, and ≥ 37 weeks).

Intrauterine growth was defined according to the INTERGROWTH-21st parameters for GA and sex [31] to classify participants into small for gestational age (SGA; birth weight < 10th centile), adequate for gestational age (AGA; birth weight 10–90 centile), and large for gestational age (LGA; birth weight > 90th centile).

### Body composition

For all the three cohorts, the outcomes were fat mass (FM) and fat-free mass (FFM) in kilograms, percentage (%FM and %FFM), and index (FMI and FFMI in kg/m^2^). We also used body mass index (BMI) in kg/m^2^ as an additional outcome. For the 2004 cohort, BMI Z-scores specific for sex and age were calculated according to the growth curves published by the World Health Organization (WHO) in 2007 [32] using ANTHRO PLUS software downloaded from the WHO website [33]. Height was measured twice by trained anthropometrists using a Harpenden metal stadiometer, with 1 mm precision (Holtain, Crymych, UK), and weight was assessed using a high precision scale (0.01 kg), part of BodPod® used for body composition assessment [34].

Body composition was evaluated by air-displacement plethysmography using BodPod® handled by specifically trained technicians. Plethysmography is a safe, fast, and noninvasive method used in various population groups (obese individuals, children, adults, and older adults) [35]. For this measure, participants remain inside the device, a closed chamber, for a few seconds without moving. It is necessary to eliminate the effect of the volume of clothing, hair, body surface, and lungs to measure with adequate accuracy and minimize disparities in body volume measurement, so all participants were provided with appropriate clothing. Sets of a rubber (swimming) cap and clothes specially made (shorts and elastane tank top) were provided to the cohorts’ participants. A predictor of the thoracic gas volume was used based on the participant’s age, sex, and height [36]. Standard equations were used to define body fat and fat-free mass at 6 [37] and 18 and 30 years of age [38].

### Covariables

Maternal covariables were collected during the mothers’ stay at the hospital. Trained interviewers recruited and interviewed the mothers and evaluated the newborns at maternity hospitals. We gathered information on maternal education level in numbers of full years at school (later categorized in 0–4, 5–8, 9–11, and ≥ 12 years), age (later categorized in < 20, 20–34, and > 34 years), smoking during pregnancy (smoking at least one cigarette per day, every day in any trimester of pregnancy - no; yes), family income during the month prior to the child’s birth, in tertiles (1st poorest; 2nd; 3rd wealthiest), and maternal pre-gestational BMI. Maternal pre-gestational BMI was classified as underweight (≤18.49 kg/m^2^), adequate (18.5 to 24.9 kg/m^2^), overweight (25.0 to 29.9 kg/m^2^), and obesity (≥30 kg/m^2^) [39].

For the participants, the covariables sex (male; female), skin color (white; non-white), birth weight (Z-score) [32], and height (cm) were employed. The hospital staff measured the child’s birth weight using electronic pediatric scales (Harpenden@) with a precision of 10 g, daily checked for accuracy by the research team. The study team measured birth length using stadiometers with an accuracy of 1 mm.

### Statistical analyses

We performed all statistical analyses using Stata version 14.0 (Stata Corp., College Station, USA). Firstly, the three cohorts were described according to maternal and child characteristics and current mean and standard deviation (SD) of FM, %FM, FMI, FFM, %FFM, FFMI, and BMI or BMI Z-score. In these analyses, chi-square and one-way ANOVA were used where appropriate. Multiple comparisons were assessed using the Bonferroni’s test.

Although there was no evidence of interaction between GA and sex among participants from the 2004 and 1993 cohorts, in the 1982 cohort the *p*-values for interaction over FM, %FM, FMI, %FFM, and BMI were 0.096, 0.104, 0.099, 0.073, and 0.115, respectively. Then, to exhibit similar data in the three cohorts, all the analyses were stratified by sex. Interaction between GA and skin color [40] was tested. However, results were non-significant for both sexes.

The strength of the association of prematurity and birth weight for gestational age with body composition and BMI was analyzed by linear regression. Beta coefficients with respective 95% confidence intervals (95%CI) for FM, FFM, %FM, %FFM, FMI, FFMI, and BMI or BMI Z-score were obtained. Adjusted hierarchical analyses were performed based on a theoretical model built by the authors. The first level was composed of maternal education, maternal age, and family income; the second level was maternal smoking during pregnancy and pre-gestational BMI; the third by skin color and birth weight (Z-score); height was included in the fourth and most proximal level. At each level, the *p*-value was verified, and those with the largest *p*-value were removed one-by-one from the model. Variables associated with the outcome at a *p*-value < 0.20 were kept in the model to control for possible confounding effects. The variables maintained for adjustment varied according to the outcome. Because participants’ height is used to construct FMI, FFMI, and BMI, this variable was not included as a confounder in the analyses for these outcomes. When exposure was weight for gestational age, birth weight was not included as a confounder in the adjusted models. The statistical analyses were performed assuming a level of significance of 5%.

Unknown GA accounted for only 0.3, 1.5% in the 2004 and 1993 cohorts, respectively. Nevertheless, for the 1982 cohort, unknown GA accounted for 21% of the sample. Due to this large loss of information, a multiple imputation process using the chained equation approach was used to impute all missing GA information, using the command *mi impute chained (reg)* in Stata. The imputation model included the variable to be imputed (GA) and the potential confounders, as described above. The stability of the imputation process was determined taking into account the average difference between the regression coefficients calculated from the complete and imputed data (bias) and the standard error of the bias. Five data sets were generated as we noted that after 5 cycles, the imputation process was found to be stable. In other words, the bias and the standard error of the bias have remained close to zero. Results for GA were calculated by averaging all five imputed data sets.

As a complementary analysis, to evaluate a possible cohort effect of GA on body composition, we assessed the difference in BMI Z-scores within the 1982, 1993, and 2004 cohorts with data gathered between 3 and 4 years of age.

### Ethical approval

The Research Ethics Committee of the Faculty of Medicine at the Federal University of Pelotas approved all visits of the Pelotas 1982, 1993, and 2004 cohorts. All methods were performed following the relevant guidelines and regulations and were approved under the protocol numbers 35/10 for the 2004 cohort (at 6 years), 05/11 for the 1993 cohort (at 18 years), and 16/12 for the 1982 cohort (at 30 years). Written informed consent was obtained from participants of the 1982 and 1993 cohorts before the interview. For the 2004 cohort participants, written informed consent was obtained from the mother or the legal guardian.

## Results

The number of live births enrolled in the cohorts was 4231 in 2004, 5249 in 1993, and 5914 in 1982. The follow-up rates at 6, 18, and 30 years in the 2004, 1993, and 1982 cohorts were 90.2, 81.3, and 63.0%, respectively.

A total of 3036 participants aged 6 years (71.8% of the original 2004 cohort), 3027 aged 18 years (57.7% of the original 1993 cohort), and 3369 aged 30 years (57.0% of the original 1982 cohort) had full information on GA and air-displacement plethysmography and were entered in the current analyses. The mean age of the 2004, 1993, and 1982 cohort members at the follow-up visit was 6.7 (SD = 0.2), 18.4 (SD = 0.3), and 30.2 (SD = 0.3) years, respectively (Supplemental Table 1). At the time of childbirth, most mothers were between 20 and 34 years old and had less than 9 years of formal education. Prevalence of maternal obesity was 11.9, 4.7, and 4.3%, and the prevalence of maternal smoking in pregnancy was 28.0, 31.8, and 34.8%, respectively, in the 2004, 1993, and 1982 cohorts. Prevalence of low birth weight (LBW; birth weight < 2500 g) was 7.7% in 2004, 7.3% in 1993, and 8.4% in 1982; prevalence of SGA children was 7.7, 14.8 and 11.4%; and prevalence of preterm birth was 13.1, 11.5 and 11.7%, respectively (Supplemental Table 1).

Supplemental Table 1 also shows the comparison between the cohort members included and not included in the current analyses. Those who died before the follow-up, as well as those who we failed to locate, were considered as losses. For the three cohorts, losses were higher among the poorest and those with LBW. In the 2004 cohort, losses were higher among children born preterm, whereas, in the 1993 cohort, the proportion of losses was higher in those born at term. In the 1993 and 1982 cohorts, losses were greater among nonwhite skin color participants, born to less educated mothers and who smoked during pregnancy. The current weight and length of cohort participants, according to gestational age, are presented in Supplemental Table 2. At 6 years, preterm boys and girls were thinner and shorter than those born at term. There was no difference in weight and height between preterm and term participants at 18 and 30 years.

Tables 1 and 2 present the mean and SD of body composition outcomes and BMI Z-scores, according to GA and sex. At 6 years of age, the means of all outcomes were lower among boys born at 34–36 weeks, in comparison to those born at term. BMI, FFM, and FFMI were also lower in boys born with ≤33 weeks than to those born at term. Among girls, the results were similar to those observed among boys, except that there was no difference in FFM and FFMI of those born at 34–36 weeks, nor in mean BMI Z-score among girls born at ≤33 weeks, in comparison to those born at term. No difference was observed among adolescents from the 1993 cohort. In the 1982 cohort, prematurity was associated with higher adiposity: men born at ≤33 and 34–36 weeks presented higher %FM and FMI and lower %FFM, compared to those born at term. There was no difference among women from the 1982 cohort.

**Table 1:** Means and standard deviations of body fat indicators according to gestational age

Gestational age		Male					Female			
		FM (kg)	FM (%)	FMI (kg/m^**2**^)	BMI^**a**^ (kg/m^**2**^)		FM (kg)	FM (%)	FMI (kg/m^**2**^)	BMI^**a**^ (kg/m^**2**^)
**6 y (2004 Cohort)**	**N**	**Mean (SD)** ***p*** **< 0.001**	**Mean (SD)** ***p*** **< 0.001**	**Mean (SD)** ***p*** **< 0.001**	**Mean (SD)** ***p*** **< 0.001**	**N**	**Mean (SD)** ***p*** **= 0.002**	**Mean (SD)** ***p*** **= 0.001**	**Mean (SD)** ***p*** **= 0.003**	**Mean (SD)** ***p*** **= 0.012**
**≤33**	36	4.8 (3.3)	19.8 (6.8)	3.2 (1.8)	0.05 (1.5)	32	5.8 (3.1)	23.9 (7.1)	4.1 (1.8)	0.51 (1.2)
**34 to 36**	167	5.1 (3.7)	20.2 (7.4)	3.4 (2.1)	0.21 (1.6)	165	5.7 (3.2)	23.2 (7.2)	3.9 (1.9)	0.40 (1.2)
**37 to 41**	1361	6.1 (3.6)	22.5 (7.9)	4.0 (2.1)	0.81 (1.5)	1275	6.8 (4.0)	25.5 (8.0)	4.6 (2.4)	0.72 (1.4)
***Total***	*1564*	*5.9 (3.6)*	*22.2 (7.9)*	*3.9 (2.1)*	*0.72 (1.5)*	*1472*	*6.6 (3.8)*	*25.2 (7.9)*	*4.5 (2.3)*	*0.67 (1.4)*
**18 y (1993 Cohort)**	**N**	**Mean (SD)** ***p*** **= 0.873**	**Mean (SD)** ***p*** **= 0.711**	**Mean (SD)** ***p*** **= 0.938**	**Mean (SD)** ***p*** **= 0.839**	**N**	**Mean (SD)** ***p*** **= 0.660**	**Mean (SD)** ***p*** **= 0.519**	**Mean (SD)** ***p*** **= 0.710**	**Mean (SD)** ***p*** **= 0.746**
**≤33**	32	12.6 (13.4)	15.7 (10.5)	4.4 (4.5)	23.4 (5.7)	42	19.6 (9.5)	31.4 (8.4)	7.6 (3.5)	23.1 (4.7)
**34 to 36**	143	12.5 (10.0)	16.5 (9.1)	4.1 (3.2)	23.2 (4.3)	133	20.9 (8.7)	32.7 (7.8)	8.1 (3.4)	23.7 (4.6)
**37 to 41**	1337	12.9 (9.5)	16.8 (8.8)	4.2 (3.1)	23.4 (4.2)	1340	20.9 (9.6)	32.8 (7.9)	8.0 (3.6)	25.5 (4.9)
***Total***	*1512*	*12.7 (9.6)*	*16.6 (8.8)*	*4.2 (3.1)*	*23.3 (4.2)*	*1515*	*20.8 (9.4)*	*32.7 (7.8)*	*8.0 (3.6)*	*23.5 (4.8)*
**30 y (1982 Cohort)**	**N**	**Mean (SD)** ***p*** **= 0.047**	**Mean (SD)** ***p*** **= 0.012**	**Mean (SD)** ***p*** **= 0.034**	**Mean (SD)** ***p*** **= 0.279**	**N**	**Mean (SD)** ***p*** **= 0.559**	**Mean (SD)** ***p*** **= 0.419**	**Mean (SD)** ***p*** **= 0.600**	**Mean (SD)** ***p*** **= 0.553**
**≤33**	4	35.0 (5.6)	37.7 (2.6)	11.6 (1.6)	30.7 (2.6)	5	23.2 (6.0)	37.2 (3.8)	9.1 (2.0)	24.2 (3.4)
**34 to 36**	141	20.8 (10.9)	24.1 (9.2)	6.8 (3.5)	26.8 (4.5)	160	26.1 (12.1)	36.6 (9.1)	10.0 (4.6)	26.3 (5.8)
**37 to 41**	1177	21.4 (11.4)	24.7 (9.0)	7.0 (3.7)	27.0 (4.9)	1157	27.0 (11.9)	37.3 (8.4)	10.3 (4.5)	26.6 (5.9)
***Total***	*1322*	*21.4 (11.3)*	*24.6 (9.0)*	*7.0 (3.7)*	*27.0 (4.9)*	*1322*	*26.8 (11.9)*	*37.2 (8.4)*	*10.3 (4.5)*	*26.5 (5.9)*

*BMI* body mass index, *SD* standard deviation, *FM* fat mass in kg, *%FM* percent of fat mass, *FMI* fat mass index in kg/m^2^

^a^At 6 years, BMI in Z-score

*P*-values calculated by Analysis of Variance (ANOVA) indicating the difference in means of body fat and BMI, between gestational age groups within each cohort

**Table 2:** Means and standard deviations of fat-free mass indicators according to gestational age

Gestational age		Male				Female		
		FFM (kg)	FFM (%)	FFMI (kg/m^**2**^)		FFM (kg)	FFM (%)	FFMI (kg/m^**2**^)
**6 y (2004 Cohort)**	**N**	**Mean (SD)** ***p*** **< 0.001**	**Mean (SD)** ***p*** **< 0.001**	**Mean (SD)** ***p*** **< 0.001**	**N**	**Mean (SD)** ***p*** **= 0.005**	**Mean (SD)** ***p*** **= 0.001**	**Mean (SD)** ***p*** **= 0.003**
**≤33**	36	17.8 (3.2)	80.2 (6.8)	7.5 (1.0)	32	17.1 (2.6)	76.0 (7.2)	7.3 (0.8)
**34 to 36**	167	18.5 (3.0)	79.7 (7.5)	7.6 (0.9)	165	17.8 (2.5)	76.8 (7.2)	7.4 (0.8)
**37 to 41**	1361	19.4 (2.8)	77.5 (7.9)	7.9 (0.9)	1275	18.3 (2.8)	74.5 (8.1)	7.6 (0.9)
***Total***	*1564*	*19.2 (2.9)*	*77.8 (7.8)*	7.9 (0.9)	*1472*	*18.2 (2.8)*	*74.8 (8.0)*	*7.5 (0.8)*
**18 y (1993 Cohort)**	**N**	**Mean (SD)** ***p*** **= 0.192**	**Mean (SD)** ***p*** **= 0.711**	**Mean (SD)** ***p*** **= 0.911**	**N**	**Mean (SD)** ***p*** **= 0.621**	**Mean (SD)** ***p*** **= 0.519**	**Mean (SD)** ***p*** **= 0.685**
**≤33**	32	57.5 (7.1)	84.3 (10.5)	3.6 (3.9)	42	40.1 (5.9)	68.6 (8.4)	6.1 (2.9)
**34 to 36**	143	57.1 (6.6)	83.5 (9.1)	3.6 (2.8)	133	40.7 (4.5)	67.3 (7.8)	6.5 (2.7)
**37 to 41**	1337	58.2 (7.4)	83.2 (8.8)	3.7 (2.7)	1340	40.3 (5.1)	67.2 (7.8)	6.5 (2.9)
***Total***	*1512*	*58.1 (7.3)*	*83.2 (8.8)*	*3.7 (2.7)*	*1515*	*40.3 (5.1)*	*67.3 (7.9)*	*6.5 (2.9)*
**30 y (1982 Cohort)**	**N**	**Mean (SD)** ***p*** **= 0.667**	**Mean (SD)** ***p*** **= 0.013**	**Mean (SD)** ***p*** **= 0.669**	**N**	**Mean (SD)** ***p*** **= 0.252**	**Mean (SD)** ***p*** **= 0.262**	**Mean (SD)** ***p*** **= 0.369**
**≤33**	4	57.6 (6.6)	62.3 (2.6)	19.1 (1.2)	5	38.4 (4.8)	62.8 (3.8)	15.1 (1.7)
**34 to 36**	141	61.2 (7.3)	75.9 (9.2)	20.0 (1.8)	160	42.5 (4.8)	63.6 (9.1)	16.2 (1.6)
**37 to 41**	1177	61.0 (8.0)	75.4 (9.0)	20.0 (2.1)	1157	42.5 (5.5)	62.5 (8.4)	16.3 (1.9)
***Total***	*1322*	*61.0 (7.9)*	*75.4 (9.1)*	*20.0 (2.0)*	*1322*	*42.5 (5.4)*	*62.6 (8.4)*	*16.2 (1.8)*

*SD* standard deviation, *FFM* fat-free mass in kg, *%FFM* percent of fat-free mass, *FFMI* fat-free mass index in kg/m^2^

*P*-values calculated by Analysis of Variance (ANOVA) indicating the difference in means of body fat and BMI, between gestational age groups within each cohort

Tables 3 and 4 show the crude and adjusted results from linear regression for males and females. Adjusted analysis for the 2004 Pelotas Cohort showed that BMI Z-score, FM, %FM, and FMI were lower in preterm boys than in boys born at term. The adjusted BMI Z-score, FM, %FM, and FMI were, respectively, − 0.48 Z-score (− 0.79; − 0.16), − 0.80 kg (− 1.45;-0.16), − 2.91 percentage points (− 4.45; − 1.36), and − 0.70 kg/m^2^ (− 1.13; − 0.28) lower in preterm boys born at 34–36 weeks of GA than among those born at term (Table 3). In the adjusted analyses for FFM, boys born at 34–36 weeks had lower FFM (− 0.4 kg; − 0.77--0.12) and FFMI (− 0.3 kg/m^2^; − 0.46--0.10) and higher %FFM (2.4; 0.87–3.90) when compared with those born at term (Table 4). In the 1993 cohort, no association was found between GA and body composition indicators or BMI (Tables 3 and 4). At 30 years, after controlling for confounders, FM (15.67 kg), %FM (13.52 percentage points), and FMI (5.11 kg/m^2^) were greater in men born at ≤33 weeks of gestation than in those born at term (Table 3). For FFM indicators, no association was found in adulthood (Table 4).

**Table 3:** Association between gestational age and body fat indicators

	FM (kg)		%FM		FMI (kg/m^**2**^)		BMI^**a**^ (kg/m^**2**^)	
	β (CI95%)		β (CI95%)		β (CI95%)		β (CI95%)	
Gestational age	Crude	Adjusted	Crude	Adjusted	Crude	Adjusted	Crude	Adjusted
**Male**								
**6 y (2004 Cohort)**	***p*** **< 0.001**	***p*** **= 0.046**	***p*** **< 0.001**	***p*** **= 0.008**	***p*** **< 0.001**	***p*** **= 0.004**	***p*** **< 0.001**	***p*** **< 0.001**
≤ 33	− 1.29 (−2.50; − 0.09)	0.22 (− 1.06; 1.51)	− 2.72 (−5.31; − 0.14)	− 0.67 (− 3.64; 2.30)	− 0.76 (− 1.47; − 0.06)	− 0.54 (− 1.38; − 0.31)	− 0,76 (− 1.26; − 0.26)	− 0.78(− 1.36; 0.20)
34 to 36	− 0.97 (− 1.54; − 0.39)	− 0.80 (− 1.45; − 0.16)	− 2.32 (− 5.31; − 0.14)	−2.39 (− 3.90; − 0.88)	− 0.57 (− 0.91; − 0.23)	−0.70 (− 1.13; − 0.27)	−0.60 (− 0.84; − 0.36)	−0.69 (− 0.99; − 0.40)
37 to 41	ref	ref	ref	ref	ref	ref	ref	ref
**18 y (1993 Cohort)**	***p*** **= 0.873**	***p*** **= 0.469**	***p*** **= 0.711**	***p*** **= 0.461**	***p*** **= 0.938**	***p*** **= 0.754**	***p*** **= 0.839**	***p*** **= 0.951**
≤ 33	−0.36 (− 3.75; 3.02)	−0.94 (− 4.81; 2.93)	− 1.16 (− 4.26; 1.94)	− 1.57 (− 4.73; 1.59)	−0.10 (− 0.63; 0.44)	−0.31 (− 1.25; 0.63)	0.02 (− 1.45; 1.48)	0.10 (− 1.75; 1.96)
34 to 36	−0.41 (− 2.08; 1.25)	0.59 (− 1.19; 2.38)	−0.32 (− 1.84; 1.20)	0.59 (− 1.05; 2.24)	− 0.01 (− 1.10; 1.08)	0.09 (− 0.40; 0.57)	− 0.22 (− 0.95; 0.51)	−0.11 (− 0.87; 0.64)
37 to 41	ref	ref	ref	ref	ref	ref	ref	ref
**30 y (1982 Cohort)**	***p*** **= 0.053**	***p*** **= 0.102**	***p*** **= 0.013**	***p*** **= 0.085**	***p*** **= 0.037**	***p*** **= 0.114**	***p*** **= 0.290**	***p*** **= 0.319**
≤ 33	13.53 (2.20; 24.87)	15.67 (0.25; 31.09)	13.13 (4.24; 22.0)	13.52 (1.17; 25.87)	4.59 (0.96; 8.22)	5.11 (0.09; 10.13)	3.68 (− 1.17; 8.54)	3.98 (− 2.64; 10.61)
34 to 36	−0.62 (− 2.64; 1.40)	−0.78 (− 2.86; 1.29)	−0.46 (− 2.04; 1.12)	−0.46 (− 2.10; 1.19)	−0.21 (− 0.85; 0.44)	−0.19 (− 0.86; 0.47)	−0.21 (− 1.08; 0.65)	−0.42 (− 1.31; 0.47)
37 to 41	ref	ref	ref	ref	ref	ref	ref	ref
**Female**								
**6 y (2004 Cohort)**	***p*** **= 0.002**	***p*** **= 0.539**	***p*** **= 0.001**	***p*** **= 0.442**	***p*** **= 0.003**	***p*** **= 0.113**	***p*** **= 0.004**	***p*** **= 0.116**
≤ 33	−1.01 (− 2.34; 0.33)	−0.16 (− 2.00; 1.69	− 1.54 (− 3.58; − 1.02)	−0.86 (− 5.04; 3.31)	−0.44 (− 1.25; 0.37)	−0.49 (− 0.99; 0.00)	−0.21 (− 0.68; 0.27)	−0.31 (− 1.03; 0.42)
34 to 36	−1.06 (− 1.69; − 0.44)	−0.40 (− 1.12; 0.31)	− 2.30 (− 4.29; 1.20)	−1.03 (− 2.66; 0.60)	−0.63 (− 1.01; − 0.26)	−0.57 (− 1.85; 0.71)	−0.32 (− 0.54; − 0.10)	−0.28 (− 0.56; 0.00)
37 to 41	ref	ref	ref	ref	ref	ref	ref	ref
**18 y (1993 Cohort)**	***p*** **= 0.666**	***p*** **= 0.554**	***p*** **= 0.519**	***p*** **= 0.418**	***p*** **= 0.710**	***p*** **= 0.442**	***p*** **= 0.746**	***p*** **= 0.440**
≤ 33	−1.34 (− 4.27; 1.58)	− 1.53 (− 4.39; 1.34)	−1.41 (− 3.83; 1.01)	−1.52 (− 4.07; 1.04)	−0.47 (− 1.58; 0.65)	− 0.62 (− 1.66; 0.43)	−0.40 (− 1.89; 1.09)	−0.76 (− 2.10; 0.58)
34 to 36	− 0.05 (− 1.74; 1.65)	0.20 (− 1.46; 1.85)	− 0.13 (− 1.53; 1.28)	0.37 (− 0.97; 1.72)	0.02 (− 0.63; 0.66)	0.14 (− 0.46; 0.74)	0.23 (− 0.63; 1.10)	0.22 (− 0.55; 1.00)
37 to 41	ref	ref	ref	ref	ref	ref	ref	ref
**30 y (1982 Cohort)**	***p*** **= 0.401**	***p*** **= 0.535**	***p*** **= 0.262**	***p*** **= 0.369**	***p*** **= 0.426**	***p*** **= 0.569**	***p*** **= 0.434**	***p*** **= 0.459**
≤ 33	−4.10 (− 14.76; 6.56)	− 2.59 (− 12.73; 7.54)	−0.37 (− 7.82; 7.09)	0.18 (− 6.95; 7.32)	−1.39 (− 5.43; 2.65)	− 1.06 (− 4.92; 2.81)	−2.55 (− 7.80; 2.70)	−2.29 (− 7.32; 2.74)
34 to 36	−1.17 (− 3.17; 0.84)	−1.07 (− 3.16; 1.01)	−1.17 (− 2.57; 0.23)	−1.04 (− 2.50; 0.41)	−0.44 (− 1.20; 0.32)	−0.37 (− 1.16; 2.81)	−0.45 (− 1.44; 0.54)	−0.49 (− 1.57; 0.59)
37 to 41	ref	ref	ref	ref	ref	ref	ref	ref

*FM* fat mass, *%FM* percentage of fat mass, *FMI* fat mass index, *BMI* body mass index

^a^At 6 years, BMI in Z-score

β refers to linear regression models. Models were adjusted for maternal (education, age, family income at birth, smoking during pregnancy, and pre-gestational BMI) and the cohort participant characteristics (birth weight

**Table 4:** Association between gestational age and fat-free mass indicators

	FFM (kg)		%FFM		FFMI (kg/m^**2**^)	
	β (CI95%)		β (CI95%)		β (CI95%)	
Gestational age	Crude	Adjusted	Crude	Adjusted	Crude	Adjusted
**Male**						
**6 y (2004 Cohort)**	***p*** **< 0.001**	***p*** **= 0.010**	***p*** **< 0.001**	***p*** **= 0.008**	***p*** **< 0.001**	***p*** **< 0.001**
≤ 33	−1.6 (− 2.50; − 0.61)	−0.5 (− 1.16; 0.12)	2.7 (0.11; 5.29)	0.7 (− 2.26; 3.67)	− 0.5 (− 0.76; − 0.17)	− 0.5 (− 0.82; − 0.13)
34 to 36	−0.8 (− 1.29; − 0.37)	− 0.4 (− 0.77; − 0.12)	2.1 (0.89; 3.40)	2.4 (0.87; 3.90)	−0.3 (− 0.42; − 0.14)	−0.3 (− 0.46; − 0.10)
37 to 41	ref	ref	ref	ref	ref	ref
**18 y (1993 Cohort)**	***p*** **= 0.192**	***p*** **= 0.684**	***p*** **= 0.711**	***p*** **= 0.454**	***p*** **= 0.911**	***p*** **= 0.562**
≤ 33	−0.7 (−3.30; 1.86)	0.4 (− 1.60; 2.43)	1.2 (− 1.94; 4.26)	1.6 (− 1.59; 4.72)	− 0.05 (− 1.01; 0.90)	−0.4 (− 1.41; 0.54)
34 to 36	−1.1 (− 2.40; 0.13)	− 0.4 (− 1.41; 0.63)	0.3 (− 1.20; 1.84)	−0.6 (− 2.17; 0.98)	−0.10 (− 0.57; 0.37)	0.1 (− 0.34; 0.63)
37 to 41	ref	ref	ref	ref	ref	ref
**30 y (1982 Cohort)**	***p*** **= 0.667**	***p*** **= 0.451**	***p*** **= 0.013**	***p*** **= 0.085**	***p*** **= 0.669**	***p*** **= 0.814**
≤ 33	−3.46 (−11.21; 4.30)	− 2.48 (− 10.95; 5.99)	−13.13 (− 22.03; − 4.24)	−13.52 (− 25.87; − 1.17)	− 0.92 (− 2.93; 1.09)	−1.01 (− 3.77; 1.75)
34 to 36	0.14 (− 1.24; 1.52)	− 0.69 (− 1.90; 0.51)	0.46 (− 1.12; 2.04)	0.46 (− 1.19; 2.10)	− 0.01 (− 0.37; 0.35)	−0.22 (− 0.62; 0.17)
37 to 41	ref	ref	ref	ref	ref	ref
**Female**						
**6 y (2004 Cohort)**	***p*** **= 0.005**	***p*** **= 0.946**	***p*** **= 0.001**	***p*** **= 0.442**	***p*** **= 0.041**	***p*** **= 0.180**
≤ 33	−1.2 (− 2.14; − 0.19)	0.03 (− 0.88; 0.95)	1.5 (− 1.29; 4.27)	0.9 (−3.1; 5.04)	− 0.3 (− 0.57; 0.04)	−0.2 (− 0.71; 0.24)
34 to 36	− 0.6 (− 1.00; − 0.10)	−0.06 (− 0.42; 0.30)	2.3 (1.03; 3.60)	1.0 (− 0.60; 2.66)	−0.1 (− 0.29; − 0.00)	−0.2 (− 0.34; 0.03)
37 to 41	ref	ref	ref	ref	ref	ref
**18 y (1993 Cohort)**	***p*** **= 0.621**	***p*** **= 0.646**	***p*** **= 0.519**	***p*** **= 0.386**	***p*** **= 0.685**	***p*** **= 0.471**
≤ 33	−0.2 (− 1.71; 1.41)	−0.2 (− 1.47; 1.15)	1.4 (− 1.01; 3.83)	1.6 (− 0.82; 3.96)	−0.40 (− 1.29; 0.50)	−0.5 (− 1.40; 0.38)
34 to 36	0.4 (− 0.47; 1.34)	0.3 (− 0.41; 1.10)	0.1 (− 1.28; 1.53)	− 0.3 (− 1.71; 1.09)	0.00 (− 0.52; 0.52)	0.1 (− 0.41; 0.63)
37 to 41	ref	ref	ref	ref	ref	ref
**30 y (1982 Cohort)**	***p*** **= 0.252**	***p*** **= 0.200**	***p*** **= 0.262**	***p*** **= 0.369**	***p*** **= 0.369**	***p*** **= 0.349**
≤ 33	−4.04 (−8.82; 073)	−3.09 (− 6.96; 0.78)	0.37 (− 7.09; 7.82)	− 0.18 (− 7.32; 6.95)	− 1.14 (− 2.73; 0.44)	− 0.90 (− 2.39; 0.59)
34 to 36	− 0.02 (− 0.92; 0.88)	− 0.39 (− 1.24; 0.45)	1.17 (− 0.23; 2.57)	1.04 (− 0.41; 2.50)	− 0.00 (− 0.30; 0.30)	−0.14 (− 0.47; 0.18)
37 to 41	ref	ref	ref	ref	ref	ref

*FFM* fat-free mass, *%FFM* percentage of fat-free mass, *FFMI* fat-free mass index

β refers to linear regression models. Models were adjusted for maternal (education, age, family income at birth, smoking during pregnancy, and pre-gestational BMI) and cohort participant characteristics (birth weight in z-score, skin color and height (except for FFMI))

The associations between weight for gestational age, body fat, and BMI are displayed in Table 5. SGA boys had less − 0.31 BMI Z-score (− 0.66; 0.03), and those born LGA had more 0.22 BMI Z-score (− 0.03; 0.47) when compared to the AGA group, but the confidence intervals included the references. As for the association between weight for gestational age and FFM indicators, Table 6 shows that at 6 years, FFM was higher among LGA boys (0.35 kg; 0.08–0.62) than in AGA boys. LGA boys and girls from the 2004 cohort had higher FFMI (0.27 kg/m^2^; 0.12–0.42 and 0.22 kg/m2; 0.06–0.37; respectively) than those born AGA. At 6 years, SGA boys had lower FFMI than boys born at term.

**Table 5:** Association between birth weight for gestational age and body fat indicators

	FM (kg)		%FM		FMI (kg/m^**2**^)		BMI^*^ (kg/m^**2**^)	
	β (CI95%)		β (CI95%)		β (CI95%)		β (CI95%)	
Gestational age	Crude	Adjusted	Crude	Adjusted	Crude	Adjusted	Crude	Adjusted
**Male**								
**6 y (2004 Cohort)**	***p*** **= 0.002**	***p*** **= 0.483**	***p*** **= 0.246**	***p*** **= 0.164**	***p*** **= 0.034**	***p*** **= 0.497**	***p*** **< 0.001**	***p*** **= 0.030**
SGA	−0.91 (− 1.58; − 2.04)	0.35 (− 0.40; 1.11)	−1.12 (− 2.57; 0.32)	1.20 (− 0.57; 2.97)	−0.43 (− 0.82; − 0.03)	−0.28 (− 0.78; 0.22)	−0.44 (− 0.71; − 0.16)	−0.31 (− 0.66; 0.03)
AGA	ref	Ref	ref	ref	ref	ref	ref	ref
LGA	0.44 (−0.03; 0.91)	−0.19 (− 0.74; 0.36)	0.24 (− 0.78; 1.27)	−0.79 (− 2.08; 0.49)	0.17 (− 0.11; 0.45)	0.06 (− 0.31; 0.43)	0.35 (0.15; 0.55)	0.22 (− 0.03; 0.47)
**18 y (1993 Cohort)**	***p*** **= 0.351**	***p*** **= 0.429**	***p*** **= 0.333**	***p*** **= 0.300**	***p*** **= 0.414**	***p*** **= 0.327**	***p*** **= 0.389**	***p*** **= 0.300**
SGA	0.43 (−1.02; 1.87)	0.31 (− 1.12; 1.74)	0.14 (− 1.18; 1.47)	0.09 (− 1.23; 1.42)	0.14 (− 0.33; 0.60)	0.14 (− 0.33; 0.61)	0.04 (− 0.13; 0.22)	0.18 (− 0.07; 0.43)
AGA	ref	Ref	ref	ref	ref	ref	ref	ref
LGA	1.00 (−0.40; 2.41)	0.91 (−0.26; 2.56)	0.97 (− 0.31; 2.26)	1.02 (− 0.27; 2.30)	0.29 (− 0.16; 0.74)	0.34 (− 0.12; 0.79)	0.12 (− 0.05; 0.29)	− 0.01 (− 0.27; 0.24)
**30 y (1982 Cohort)**	***p*** **= 0.999**	***p*** **= 0.343**	***p*** **= 0.698**	***p*** **= 0.754**	***p*** **= 0.959**	***p*** **= 0.332**	***p*** **= 0.946**	***p*** **= 0.356**
SGA	0.01 (−2.04; 2.06)	1.67 (− 0.71; 4.06)	−0.12 (− 1.74; 1.50)	0.08 (−1.63; 1.79)	0.09 (− 0.57; 0.74)	0.54 (− 0.23; 1.31)	0.13 (− 0.74; 1.00)	0.73 (− 0.29; 1.74)
AGA	ref	Ref	ref	ref	ref	ref	ref	ref
LGA	0.00 (−1.75; 1.76)	0.70 (− 1.30; 2.70)	0.13 (− 1.25; 1.52)	0.56 (− 0.90; 2.02)	0.05 (− 0.51; 0.61)	0.25 (− 0.40; 0.89)	− 0.05 (− 0.79; 0.70)	0.21 (− 0.64; 1.06)
**Female**								
**6 y (2004 Cohort)**	***p*** **< 0.001**	***p*** **= 0.934**	***p*** **= 0.011**	***p*** **= 0.517**	***p*** **< 0.001**	***p*** **= 0.612**	***p*** **< 0.001**	***p*** **= 0.351**
SGA	−0.94 (−1.70; − 0.18)	0.20 (− 0.93; 1.33)	−1.44 (− 3.01; 0.14)	1.46 (− 1.11; 4.03)	−0.53 (− 0.99; − 0.07)	0.40 (− 0.39; 1.19)	− 0.41 (− 0.67; − 0.14)	0.32 (− 0.21; 0.77)
AGA	ref	Ref	ref	ref	ref	ref	ref	ref
LGA	0.93 (0.41; 1.45)	−0.02 (− 0.87; 0.83)	1.18 (0.11; 2.26)	− 0.65(− 2.58; 1.28)	0.45 (0.14; 0.76)	− 0.11 (− 0.70; 0.48)	0.34 (0.16; 0.52)	− 0.13 (− 0.47; 0.21)
**18 y (1993 Cohort)**	***p*** **= 0.100**	***p*** **= 0.719**	***p*** **= 0.170**	***p*** **= 0.697**	***p*** **= 0.110**	***p*** **= 0.633**	***p*** **= 0.219**	***p*** **= 0.438**
SGA	1.29 (−0.03; 2.61)	0.46 (−1.43; 2.36)	0.95 (− 0.15; 2.0)	0.06 (− 1.51; 1.63)	0.50 (− 0.00; 1.00)	0.23 (− 0.49; 0.95)	0.15 (− 0.02; 0.32)	0.12 (− 0.12; 0.36)
AGA	ref	Ref	ref	ref	ref	ref	ref	ref
LGA	−0.49 (−1.94; 0.87)	0.40 (−1.67; 2.48)	− 0.32 (− 1.53; 0.89)	0.60 (− 1.08; 2.36)	− 0.14 (− 0.69; 042)	0.15 (− 0.63; 0.94)	0.01 (− 0.17; 0.20)	0.05 (− 0.22; 0.31)
**30 y (1982 Cohort)**	***p*** **= 0.046**	***p*** **= 0.065**	***p*** **= 0.093**	***p*** **= 0.999**	***p*** **= 0.056**	***p*** **= 0.900**	***p*** **= 0.093**	***p*** **= 0.883**
SGA	1.12 (−0.92; 3.16)	1.29 (− 0.83; 3.41)	0.70 (− 0.71; 2.12)	0.07 (− 1.54; 1.68)	0.53 (− 0.24; 1.30)	0.15 (− 0.50; 0.80)	0.71 (− 0.30; 1.72)	0.20 (− 0.68; 1.07)
AGA	ref	Ref	ref	ref	ref	ref	ref	ref
LGA	−1.95 (−3.82; 3.16)	− 1.78 (− 3.72; 0.15)	−1.18 (−2.47; 0.12)	0.09 (− 1.29; 1.46)	− 0.64 (− 1.35; 0.07)	0.03 (− 0.52; 0.59)	− 0.69 (− 1.62; 0.24)	−0.06 (− 0.81; 0.68)

*FM* fat mass, *%FM* percentage of fat mass, *FMI* fat mass index, *BMI* body mass index, *SGA* small for gestational age, *AGA* adequate for gestational age, *LGA* large for gestational age. At 6 years, BMI in Z-score

β refers to linear regression models. Models were adjusted for maternal (education, age, family income at birth, smoking during pregnancy, and pre-gestational BMI) and the cohort participant characteristics (skin color and height (except for FMI and BMI))

**Table 6:** Association between birth weight for gestational age and fat-free mass indicators

	FFM (kg)		%FFM		FFMI (kg/m^**2**^)	
	β (CI95%)		β (CI95%)		β (CI95%)	
Gestational age	Crude	Adjusted	Crude	Adjusted	Crude	Adjusted
**Male**						
**6 y (2004 Cohort)**	***p*** **< 0.001**	***p*** **= 0.031**	***p*** **= 0.246**	***p*** **= 0.175**	***p*** **< 0.001**	***p*** **< 0.001**
SGA	−1.39 (− 1.91; −0.87)	−0.09 (− 0.46; 0.29)	1.12 (− 0.32; 2.57)	−1.07 (− 0.43; 2.13)	−0.39 (− 0.55; − 0.23)	−0.40 (− 0.60; − 0.20)
AGA	ref	ref	ref	ref	ref	ref
LGA	1.10 (0.73; 1.47)	0.35 (0.08; 0.62)	−0.24 (−1.27; 0.78)	0.85 (− 0.43; 2.13)	0.34 (0.23; 0.46)	0.27 (0.12; 0.42)
**18 y (1993 Cohort)**	***p*** **= 0.181**	***p*** **= 0.496**	***p*** **= 0.333**	***p*** **= 0.121**	***p*** **= 0.380**	***p*** **= 0.208**
SGA	0.99 (−0.11; 2.09)	0.47 (−0.40; 1.34)	− 0.14 (−1.47; 1.18)	−0.02 (− 1.37; 1.33)	0.12 (− 0.29; 0.53)	0.06 (− 0.35; 0.48)
AGA	ref	ref	ref	ref	ref	ref
LGA	0.45 (−0.62; 1.53)	−0.15 (− 0.99; 0.69)	−0.97 (− 2.26; 0.31)	−1.36 (− 2.67; 0.05)	0.27 (− 0.13; 0.67)	0.37 (− 0.04; 0.77)
**30 y (1982 Cohort)**	***p*** **= 0.327**	***p*** **= 0.599**	***p*** **= 0.968**	***p*** **= 0.754**	***p*** **= 0.774**	***p*** **= 0.697**
SGA	−0.54 (−1.95; 0.86)	0.63 (− 0.60; 1.86)	0.12 (− 1.50; 1.74)	−0.08 (− 1.79; 1.63_	0.03 (− 0.33; 0.40)	0.17 (− 0.23; 0.57)
AGA	ref	ref	ref	ref	ref	ref
LGA	−0.85 (− 2.05; 0.35)	0.16 (− 0.92; 1.24)	−0.13 (−1.52; 1.25)	−0.56 (− 2.02; 0.90)	−0.10 (− 0.41; 0.21)	0.02 (− 0.33; 0.37)
**Female**						
**6 y (2004 Cohort)**	***p*** **< 0.001**	***p*** **= 0.633**	***p*** **= 0.011**	***p*** **= 0.738**	***p*** **< 0.001**	***p*** **= 0.010**
SGA	−1.03 (− 1.58; −0.49)	−0.17 (− 0.63; 0.29)	1.44 (− 0.14; 3.01)	−0.75 (− 2.81; 1.30)	−0.33 (− 0.50; − 0.15)	−0.13 (− 0.37; 0.10)
AGA	ref	ref	ref	ref	ref	ref
VLGA	0.96 (0.59; 1.33)	0.08 (−0.22; 0.39)	−1.18 (− 2.26; − 0.11)	0.16 (− 1.21; 1.53)	0.28 (0.17; 0.40)	0.22 (0.06; 0.37)
**18 y (1993 Cohort)**	***p*** **= 0.361**	***p*** **= 0.411**	***p*** **= 0.170**	***p*** **= 0.348**	***p*** **= 0.102**	***p*** **= 0.267**
SGA	0.47 (−0.24; 1.17)	0.36 (−0.23; 0.94)	− 0.95 (−2.04; 0.15)	−0.67 (−1.75; 0.40)	0.40 (− 0.00; 0.81)	0.30 (− 0.10; 0.70)
AGA	ref	ref	ref	ref	ref	ref
LGA	−0.15 (− 0.93; 0.63)	0.26 (− 0.39; 0.91)	0.32 (− 0.89; 1.53)	0.34 (− 0.85; 1.54)	−0.13 (− 0.58; 0.32)	−0.11 (− 0.56; 0.33)\
**30 y (1982 Cohort)**	***p*** **= 0.512**	***p*** **= 0.698**	***p*** **= 0.093**	***p*** **= 0.132**	***p*** **= 0.435**	***p*** **= 0.679**
SGA	0.08 (−0.85; 1.01)	0.37 (−0.49; 1.22)	− 0.70 (−2.12; 0.71)	−0.15 (−1.65; 1.34)	0.18 (− 0.12; 0.49)	0.15 (− 0.18; 0.47)
AGA	ref	ref	ref	ref	ref	ref
LGA	−0.48 (−1.33; 0.37)	0.11 (− 0.67; 0.89)	1.18 (− 0.12; 2.47)	1.34 (− 0.01; 2.69)	−0.05 (− 0.33; 0.23)	0.02 (− 0.27; 0.32)

*FFM* fat-free mass, *%FFM* percentage of fat-free mass, *FFMI* fat-free mass index, *SGA* small for gestational age, *AGA* adequate for gestational age, *LGA* large for gestational age

β refers to linear regression models. Models were adjusted for maternal (education, age, family income at birth, smoking during pregnancy, and pre-gestational BMI) and the cohort participant characteristics (skin color and height (except for FMI and BMI))

The complementary analyses showed that in the 2004 cohort, at 4 years of age, BMI Z-scores increased with GA only in boys (*p* < 0.001), also present among males at 3 years of age in the 1982 cohort. At 4 years in the 1993 cohort, there was no association between GA and BMI Z-scores in the adjusted analyses (Supplemental Table 3).

## Discussion

The present study investigated the association of prematurity with body composition and BMI at childhood, adolescence, and adulthood using three different population-based cohort studies. Results suggest that prematurity is associated with lower body composition in childhood but with higher adiposity in adulthood. In the 6-year-old boys FM, %FM, FMI, BMI Z-score, FFM, and FFMI remained lower among those born at 34–36 weeks of GA after adjustment for confounders. In the 1982 Cohort, an inverse association was observed: men born at ≤33 weeks of GA presented higher FM, %FM, and FMI than those born at term. No association of preterm birth and weight for gestational age with body composition or BMI was observed in female participants in the three cohorts nor in adolescent males (1993 cohort).

The plausibility of the association between preterm birth and less body fat in childhood relies on the fact that energy (fat and glycogen) and nutrient storage in the fetus occur mainly in the last trimester of gestation, leading to low energy and nutrient reserve in preterm newborns [41, 42]. Preterm newborns also grow differently in the first months of life, and weight loss is inversely proportional to GA and directly proportional to the duration of clinical intercurrences and intrauterine nutritional restriction [18]. Additionally, preterm infants have difficulty absorbing fatty acids due to functional immaturity of the gastrointestinal tract [43].

Our findings in the 2004 cohort suggest that the reduced fat content at birth persists throughout childhood. Means of FM in 34–36 weeks of gestation were closer to those seen in preterm children born at ≤33 weeks than those born at term, consistent with the disadvantaged nutritional status described in these children [8]. Our results are consistent with data from children aged 8–12 years in the United Kingdom [20], in which boys and girls born preterm were lighter and presented lower FM than those born at term. On the other hand, Piemontese et al. [18] and Johnson et al. [44] showed that infants born preterm had significantly greater total body fat in term equivalent age, and Scheurer et al. [21] reported no association between prematurity (< 35 weeks of GA) and body fat at 4 years of age in both sexes. The usual nutrient acquisition of the fetus during the last trimester of pregnancy is higher than in any other period of life, with marked FM and FFM deposition and hepatic storage of micronutrient reserves, such as iron, zinc, and copper [44]. The mechanisms responsible for the difference in body composition of preterm infants and the relative deficit of FFM are likely to be multifactorial. They may include the availability of nutrients for growth and other factors that influence the handling or availability of nutrients, such as concurrent illness (infection and lung disease) hormonal influences (use of postnatal corticosteroids) [44].

Other factors may play a role in the child body composition, such as maternal complications (gestational hypertension and gestational diabetes), errors in the estimation of GA, elective C-section, and poor-quality or late ultrasound evaluation. The latter factor contributes to the physician’s decision to induce or not tocolysis or perform a caesarean section before completing pregnancy [45]. The GA can be overestimated up to 1.8 weeks by inaccurate ultrasound as described elsewhere [45]. C-section prevalence in the 2004 cohort (45.2%) almost doubled concerning the 1982 cohort (27.7%) [46]. Although the information on elective C-sections is complicated to obtain from hospital records as physicians are reluctant to admit that the operation did not have a medical indication [47], the causes of prematurity in 2004 could be due to the medicalization of childbirth and not to biological factors. Thus, the association between GA and body composition in childhood observed in the 2004 cohort could be due to the result of a cohort effect rather than a biological effect of GA. However, the complementary analyses showed that the association between GA and BMI Z-score observed in the 2004 cohort at 6 years was also present among males at 3 years of age in the 1982 cohort. It indicates that this association was due to GA and not to a cohort effect.

We found no association between prematurity and body composition among adolescents from the 1993 cohort. Similarly, a study carried out in Spain evaluating body composition in childhood and adolescence using Dual-energy X-ray absorptiometry found no difference between those born preterm and those born at term [48]. Similarly, Kaczmarczy et al. [17] evaluated body composition using electrical bioimpedance in adolescent girls (10–14 years) in the United States. They did not detect any difference between those born preterm or at term.

The results from the 1982 cohort (30 years) pointed out higher body fat content in men born at ≤33 weeks of GA than in those born at term. Our results are consistent with other studies conducted in the United Kingdom [49], Holland [50], and Finland [51], that found increased total body fat and higher abdominal fat among those born < 32 weeks [50], < 34 weeks [49, 51], and 34–36 weeks of GA [51]. .Mathai et al. [52], in a cohort study from New Zealand, reported that total body fat, truncal body fat, and the android-to-gynoid fat ratio at 30 years were higher among those born at a mean of 33.3 weeks of GA, in comparison to those born at term. Additionally, preterm cohort participants’ offspring who were born at term and were between 5 and 10 years old tended to have more body fat, higher truncal fat, and android-to-gynoid fat ratio than term offspring from term cohort participants, thus suggesting that negative consequences of preterm birth over body composition may extend to the subsequent generation [52]. A pooled analysis of data from four Nordic Nations found a higher risk of young adult death from chronic diseases (obesity is among the main risk factors) [53]. The individuals from these studies were born moderate preterm and earlier (23–33 weeks), late preterm (34–36 weeks), and early-term (37–38 weeks) compared with those born at full term [53].

In our study, the interaction between GA and sex over body fat indicators at age 30 years may explain the observed association among men and the lack of association among women at the three cohorts. There is evidence that exposure to exogenous and endogenous changes during specific windows of developmental programming may affect the susceptibility to chronic non-communicable diseases of the offspring, with a difference between males and females regarding age of onset and severity of disease outcomes [54].

Mechanisms underlining the association of GA with body composition in childhood and adult life are not fully established. Our theoretical model of determination, current schooling, alcohol consumption, physical activity, and eating habits were potential mediators or effect modifiers in the association between GA and body composition. Although studies showing these mechanisms have not been found, we believe that at least part of the body composition is due to these relationships.

This study has strengths and limitations. The strengths include the large sample size of three population-based birth cohorts at three different life stages in a setting where preterm births and excessive weight rates in the population are high [18, 50]. Besides having six indirect body composition indicators obtained by air-displacement plethysmography, we evaluated the double-indirect indicator BMI, aiming to facilitate comparability with other studies and communication of our findings. Moreover, all data were collected by employing standardized methods by trained field workers. Finally, we were able to use prospectively measured variables from early life to adjust for confounding effects.

Due to temporal trends and more interventionist medicine over the years [55], an increase in preterm birth prevalence over 22 years (from 1982 to 2004) was registered in Pelotas [56]. Despite this fact, the role of the method for the assessment of GA must be considered when interpreting our findings. In 1982, only the date of the last menstrual period was employed for determining GA. However, for both 1993 and 2004, the algorithm proposed by the NCHS was used [28]. So, the prevalence of preterm birth in 1982 was probably underestimated. It is highly likely that at least some 30-year-old men of unknown GA belonged to the preterm group, affecting the precision of our estimates. Even with the multiple imputation process for the GA variable in the 1982 cohort, this limitation must be considered. Additionally, as plethysmography was not available in our laboratory before 2010, it was not possible to assess body composition at similar ages in the three cohorts. Finally, we had a large loss of information for GA in the 1982 cohort, possibly due to the lack of neonatal technology in the 1980s, decreasing the statistical power of our analyses. However, through multiple imputations, we tried to lessen this problem. Furthermore, we could not assess the contribution of spontaneous or medically indicated preterm birth over body composition.

Lower neonatal and childhood survival in the 1982 cohort may have introduced a survivor bias, with only the healthiest reaching adulthood [57]. On the other hand, social conditions, maternal health, and neonatal care and treatment have changed substantially between 1982 and 2004 in Pelotas [45]*.* Therefore, it is unclear to what extent our findings on body composition at adulthood are generalizable to children born today and to children in other settings with different resources. It remains to be observed whether higher FM in adult individuals born before term, as observed at the 1982 cohort, will apply to the 2004 cohort participants when they reach 30 years old. Body composition outcomes in the 2004 cohort could potentially be worse owing to the survival of frailer infants born at earlier gestational ages. In contrast, other outcomes could be better due to improved follow-up care.

## Conclusions

Preterm born individuals present lower FM and FFM at 6 years of age and higher FM at 30 years than those born full term, but such association seems to be sex-dependent, and it is significant only among males.

## Supplementary Information

**Additional file 1: Supplemental Table 1.** Characteristics of mothers and participants of The Pelotas 2004, 1993 and 1982 Birth Cohorts.

**Additional file 2: Supplemental Table 2.** Mean weight and height, according to gestational age.

**Additional file 3: Supplemental Table 3.** Mean (standard deviation) body mass index Z-scores (childhood) according to gestational age.

## Data Availability

The datasets used and analyzed during the current study are available from the corresponding author on reasonable request. Applications to use the data should be made by contacting the researchers of the Pelotas cohorts and filling the application form for the Pelotas Birth Cohorts available at [http://www.epidemio-ufpel.org.br/site/content/estudos/formularios.php].

## Abbreviations

DoHaD: Developmental Origins of Health and Disease
GA: Gestational age
NCHS: National Center for Health Statistics
FM: Fat mass in kilograms
%FM: Percentage of fat mass
FMI: Fat mass index in kg/m^2^
FFM: Fat-free mass in kilograms
%FFM: Percentage of fat-free mass
FMI: Fat-free mass index in kg/m^2^
BMI: Body mass index in kg/m^2^ at age 18 and 30 years, and BMI Z-score at 6 years
WHO: World Health Organization
SD: Standard deviation
95%CI: 95% confidence intervals
