# Supplementary material for: Prematurity and body composition at 6, 18, and 30 years of age: Pelotas (Brazil) 2004, 1993, and 1982 birth cohorts
Source: BMC Public Health. 2021 Feb 9;21:321. doi: 10.1186/s12889-021-10368-w (PMC7871570; doi:10.1186/s12889-021-10368-w)
Supplement: Supplementary file 1 — Additional file 1: Supplemental Table 1. Characteristics of mothers and participants of The Pelotas 2004, 1993 and 1982 Birth Cohorts. [file 12889_2021_10368_MOESM1_ESM.docx]

**Supplemental Table 1.** Characteristics of mothers and participants of The Pelotas 2004, 1993 and 1982 Birth Cohorts.

|  | | **2004 Cohort** | | **1993 Cohort** | | **1982 Cohort** | |
| --- | --- | --- | --- | --- | --- | --- | --- |
|  | | **Not Included in the current analyses** | **Included in the current analyses** | **Not Included in the current analyses** | **Included in the current analyses** | **Not Included in the current analyses** | **Included in the current analyses** |
| **Characteristics** | | **N=1195** | **N=3036** | **N=2222** | **N=3027** | **N=2545** | **N=3369** |
|  |  |  | **N (%)** |  | **N (%)** |  | **N (%)** |
| **Maternal education (full years)** | | p=0.059 | | p<0.001 | | p=0.029^2^ | |
| 0 to 4 | | 209 (17.8) | 445 (14.8) | 710 (32.0) | 758 (25.1) | 892 (35.1) | 1068 (31.7) |
| 5 to 8 | | 476 (40.5) | 1255 (41.7) | 997 (44.9) | 1427 (47.2) | 1019 (40.1) | 1435 (42.6) |
| 9 to 11 | | 366 (31.1) | 1015 (33.7) | 350 (15.8) | 573 (19.0) | 288 (11.3) | 366 (10.9) |
| ≥12 | | 125 (10.6) | 295 (9.8) | 163 (7.3) | 264 (8.7) | 343 (13.5) | 496 (14.7) |
| **Maternal age** | | p=0.134 | | p=0.056 | | p=0.268 | |
| <20 | | 219 (18.4) | 580 (19.1) | 406 (18.3) | 509 (16.8) | 406 (16.0) | 506 (15.0) |
| 20 to 34 | | 832 (69.8) | 2033 (67.0) | 1595 (71.8) | 2161 (71.4) | 1873 (73.6) | 2542 (75.5) |
| ≥35 | | 141 (11.8) | 422 (13.9) | 220 (9.9) | 357 (11.8) | 265 (10.4) | 321 (9.5) |
| **Pre-gestational BMI** | | p=0.003 | | p=0.267 | | p=0.781 | |
| Underweight (≤18.49 kg/m^2^) | | 194 (23.5) | 405 (18.9) | 192 (9.1) | 259 (8.7) | 174 (8.2) | 214 (7.5) |
| Adequate (18.5-24.9 kg/m^2^) | | 399 (48.4) | 1023 (56.9) | 1481 (69.9) | 2040 (68.5) | 1475 (69.8) | 2023 (70.7) |
| Overweight (25.0-29.9 kg/m^2^) | | 149 (18.1) | 503 (28.0) | 340 (16.1) | 540 (18.1) | 372 (17.6) | 503 (17.6) |
| Obesity (≥30 kg/m^2^) | | 83 (10.1) | 214 (11.9) | 106 (5.0) | 139 (4.7) | 93 (4.4) | 122 (4.3) |
| **Maternal smoking during pregnancy** | | p=0.530 | | p=0.003 | | p=0.154 | |
| No | | 857 (71.8) | 2210 (72.8) | 1431 (64.4) | 2066 (68.3) | 1614 (63.4) | 2197 (65.2) |
| Yes | | 336 (28.2) | 826 (28.0) | 791 (35.6) | 961 (31.8) | 931 (36.6) | 1172 (34.8) |
| **Family income at birth (tertiles)** | | p=0.010 | | p<0.001 | | p<0.001 | |
| 1° (poorest) | | 443 (37.1) | 986 (32.5) | 1003 (46.6) | 1233 (41.0) | 937 (36.8) | 1026 (30.5) |
| 2° | | 365 (30.6) | 1039 (34.2) | 584 (27.1) | 861(28.9) | 806 (31.7) | 1173 (34.8) |
| 3° (wealthiest) | | 385 (32.3) | 1011 (33.3) | 567 (26.3) | 899 (30.1) | 802 (31.5) | 1170 (34.7) |
|  |  |  |  | | | | |
|  |  |  | ***Participants characteristics*** | | | | |
| **Sex** | | p=0.450 | | p=0.553 | | p=0.669 | |
| Male | | 631 (52.8) | 1564 (51.5) | 1091 (49.1) | 1512 (49.9) | 1299 (51.0) | 1738 (51.6) |
| Female | | 564 (47.2) | 1472 (48.5) | 1130 (50.6) | 1515 (50.1) | 1246 (49.0) | 1630 (48.4) |
| **Skin color** | | p=0.807 | | p=0.040 | | p= 0.667 | |
| White | | 659 (2067) | 2067 (68.1) | 876 (61.9) | 1893 (65.1) | 1362 (75.0) | 1876 (75.6) |
| Non-white | | 303 (31.5) | 969 (31.9) | 539 (38.1) | 1015(34.9) | 453 (25.0) | 605 (24.4) |
| **Birth weight (g)** | | p<0.001 | | p<0.001 | | p=0.001 | |
| <2.500 | | 189 (15.9) | 234 (7.7) | 289 (13.1) | 221 (7.3) | 253 (10.0) | 281 (8.4) |
| 2.500- 2.999 | | 295 (24.8) | 747 (24.6) | 557 (25.3) | 754 (24.9) | 650 (25.6) | 743 (22.1) |
| 3.000 - 3.499 | | 445 (37.4) | 1206 (39.7) | 844 (38.3) | 1206 (39.9) | 933 (36.7) | 1287 (38.2) |
| 3.500 - 3.999 | | 212 (17.8) | 700 (23.1) | 397 (18.0) | 684 (22.6) | 578 (22.7) | 839 (24.9) |
| ≥4.000 | | 49 (4.1) | 149 (4.9) | 119 (5.4) | 161 (5.3) | 129 (5.1) | 216 (6.4) |
| **Weight for gestational age** | | p<0.001 | | p<0.001 | | p<0.001 | |
| SGA | | 125 (12.0) | 233 (7.7) | 296 (20.7) | 447 (14.8) | 257 (13.2) | 298 (11.4) |
| AGA | | 769 (74.0) | 2265 (74.1) | 972 (68.0) | 2171 (71.4) | 1462 (75.1) | 1915 (73.4) |
| LGA | | 145 (14.0) | 538 (17.7) | 162 (11.3) | 408 (13.5) | 229 (11.8) | 397 (15.2) |
| **Gestational age (weeks)** | | p<0.001 | | p<0.001 | | p<0.001 | |
| ≤33 | | 72 (7.9) | 68 (2.2) | 69 (5.8) | 74 (2.4) | 40 (2.4) | 9 (0.3) |
| 34 - 36 | | 140 (15.4) | 332 (10.9) | 153 (12.9) | 276 (9.1) | 56 (3.4) | 301 (11.4) |
| 37 - 41 | | 700 (76.8) | 2636 (86.8) | 960 (81.2) | 2677 (88.5) | 1570 (94.2) | 2334 (88.3)40 |

BMI, body mass index. SGA, small for gestational age. AGA, adequate for gestational age. LGA, large for gestational age.

P-values calculated by Chi-square test.
