# Supplementary material for: Prematurity and body composition at 6, 18, and 30 years of age: Pelotas (Brazil) 2004, 1993, and 1982 birth cohorts
Source: BMC Public Health. 2021 Feb 9;21:321. doi: 10.1186/s12889-021-10368-w (PMC7871570; doi:10.1186/s12889-021-10368-w)
Supplement: Supplementary file 2 — Additional file 2: Supplemental Table 2. Mean weight and height, according to gestational age. [file 12889_2021_10368_MOESM2_ESM.docx]

**Supplemental Table 2.** Mean weight and height, according to gestational age.

| **Gestational age** | **Male** | | | **Female** | | | **Total** |  |
| --- | --- | --- | --- | --- | --- | --- | --- | --- |
|  | **Total N** | **Weight (kg)** | **Height (cm)** | **Total N** | **Weight (kg)** | **Height (cm)** | **Weight (kg)** | **Height (cm)** |
|  |  | **Mean (SD)** | **Mean (SD)** |  | **Mean (SD)** | **Mean (SD)** | **Mean (SD)** | **Mean (SD)** |
| ***2004 Cohort*** |  |  |  |  |  |  |  |  |
| **6 years** | **N** | **p<0.001** | **p<0.001** | **N** | **p=0.001** | **p<0.001** | **p<0.001** | **p<0.001** |
| **≤33** | 36 | 22.6 (6.0**)** | 118.8 (7.1) | 32 | 23.0 (5.4) | 116.9 (5.5) | 22.8 (5.7) | 117.9 (6.4) |
| **34 to 36** | 167 | 23.7 (6.1) | 120.8 (5.8) | 165 | 23.5 (5.2) | 119.3 (5.7) | 23.6 (5.7) | 120.0 (5.8) |
| **37 to 41** | 1361 | 25.4 (5.8) | 121.8 (5.5) | 1275 | 25.1 (6.2) | 120.6 (5.6) | 25.3 (6.0) | 121.2 (5.5) |
| ***Total*** | 1564 | 25.2 (5.9) | 121.6 (5.6) | *1472* | 24.9 (6.1) | *120.4 (5.6)* | *25.0 (6.0)* | *121.0 (5.6)* |
| ***1993 Cohort*** |  |  |  |  |  |  |  |  |
| **18 years** | **N** | **p=0.446** | **p=0.061** | **N** | **p=0.721** | **p=0.755** | **p=0.478** | **p=0.100** |
| **≤33** | 32 | 70.0 (17.7) | 171.8 (6.2) | 42 | 59.7 (13.5) | 160.5 (6.5) | 64.2 (16.2) | 165.4 (8.4) |
| **34 to 36** | 143 | 69.6 (14.1) | 173.2 (6.6) | 133 | 61.6 (11.9) | 161.1 (5.8) | 65.7 (13.6) | 167.4 (8.7) |
| **37 to 41** | 1337 | 71.1 (14.4) | 174.2 (7.0) | 1340 | 61.2 (13.3) | 161.3 (6.4) | 66.1 (14.7) | 167.7 (9.3) |
| ***Total*** | 1512 | 70.9 (14.5) | 174.0 (7.0) | *1515* | 61.2 (13.2) | *161.2 (6.4)* | *66.1 (14.7)* | *167.6 (9.3)* |
| ***1982 Cohort*** |  |  |  |  |  |  |  |  |
| **30 years** | **N** | **p=0.456** | **p=0.871** | **N** | **p=0.371** | **p=0.796** | **p=0.480** | **p=0.660** |
| **≤33** | 4 | 92.5 (11.4) | 173.6 (7.9) | 5 | 61.6 (10.5) | 159.8 (6.1) | 75.4 (19.2) | 165.9 (11.6) |
| **34 to 36** | 141 | 82.0 (15.0) | 174.9 (6.9) | 160 | 68.6 (15.1) | 161.7 (5.9) | 74.8 (16.6) | 167.8 (9.2) |
| **37 to 41** | 1177 | 82.4 (16.9) | 174.6 (6.8) | 1157 | 69.8 (16.3) | 161.6 (6.1) | 76.2 (17.7) | 168.2 (9.2) |
| ***Total*** | *1322* | *82.4 (16.7)* | *174.6 (6.8)* | *1322* | *69.6 (16.2)* | *161.6 (6.1)* | *76.0 (17.6)* | *168.1 (9.2)* |

SD: standard deviation

P-value based on Analysis of Variance (ANOVA)
