# Supplementary material for: Prematurity and body composition at 6, 18, and 30 years of age: Pelotas (Brazil) 2004, 1993, and 1982 birth cohorts
Source: BMC Public Health. 2021 Feb 9;21:321. doi: 10.1186/s12889-021-10368-w (PMC7871570; doi:10.1186/s12889-021-10368-w)
Supplement: Supplementary file 3 — Additional file 3: Supplemental Table 3. Mean (standard deviation) body mass index Z-scores (childhood) according to gestational age. [file 12889_2021_10368_MOESM3_ESM.docx]

**Supplemental Table 3**. Mean (standard deviation) body mass index Z-scores (childhood) according to gestational age.

| **Gestational age** | **Male BMI (kg/m^2^)** | | | **Female BMI (kg/m^2^)** | | |
| --- | --- | --- | --- | --- | --- | --- |
|  | **Mean BMI Z-score (SD)** | **β (CI95%)** | **β (CI95%)** | **Mean BMI Z-score (SD)** | **β (CI95%)** | **β (CI95%)** |
|  |  | **Crude** | **Adjusted** |  | **Crude** | **Adjusted** |
| **3 years (1982 Cohort)** | **p=0.041** | **p=0.041** | **p=0.052** | **p=0.839** | **p=0.839** | **p=0.885** |
| **≤33** | 1.5 (1.1) | 0.87 (-0.30; 2.04) | 0.75 (-0.41; 1.92) | 0.9 (0.5) | 0.10 (-0.80; 1.00) | 0.08 (-0.83; 0.99) |
| **34 to 36** | 0.4 (0.9) | -0.21 (-0.42; -0.01) | -0.22 (-0.42; -0.01) | 0.4 (0.9) | -0.05 (-0.24; 0.14) | -0.05 (-0.25; 0.15) |
| **37 to 41** | 0.6 (1.0) | ref. | ref. | 0.6 (1.0) | ref. | ref. |
| **Total** | 0.6 (1.0) |  |  | 0.6 (1.0) |  |  |
| **4 years (1993 Cohort)** | **p=0.291** | **p=0.290** | **p=0.371** | **p=0.100** | **p=0.100** | **p=0.071** |
| **≤33** | 0.9 (1.7) | 0.24 (-0.38; 0.86) | 0.65 (-0.38; 1.67) | -0.02 (0.9) | -0.50 (-0.97; -0.04) | -0.4319 (-0.80; -0.06) |
| **34 to 36** | 0.4 (1.1) | -0.24 (-0.61; 0.12) | -0.11 (-0.49; 0.26) | 0.5 (1.6) | 0.03 (-0.30; 0.37) | -0.03 (-0.45; 0.38) |
| **37 to 41** | 0.7 (1.3) | ref. | ref. | 0.5 (1.2) | ref. | ref. |
| **Total** | 0.6 (1.3) |  |  | 0.4 (1.2) |  |  |
| **4 years (2004 Cohort)** | **p<0.001** | **p<0.001** | **p<0.001** | **p=0.024** | **p=0.024** | **p=0.234** |
| **≤33** | 0.3 (1.5) | -0.53 (-0.92; -0.14) | -0.79 (-1.29; -0.29) | 0.5 (1.0) | -0.25 (-0.66; 0.16) | -0.28 (-0.93; 0.38) |
| **34 to 36** | 0.5 (1.3) | -0.44 (-0.63; -0.25) | -0.50 (-0.76; -0.25) | 0.5 (1.1) | -0.27 (-0.47; -0.06) | -0.21 (-0.47; 0.06) |
| **37 to 41** | 0.9 (1.3) | ref. | ref. | 0.8 (1.4) | ref. | ref. |
| ***Total*** | *0.83 (1.3)* |  |  | *0.72 (1.3)* |  |  |

BMI: body mass index in Z-score. SD: standard deviation

β refers to linear regression models. Models were adjusted for maternal (education, age, family income at birth, smoking during pregnancy, and pre-gestational BMI) and the cohort participant characteristics (birth weight in z-score and skin color.
